# Subnipple Cyst Formation Is an Important Diagnostic Clue for Mixed-Type Squamous Cell Carcinomas of the Breast: A Case Report

**DOI:** 10.7759/cureus.93978

**Published:** 2025-10-06

**Authors:** Togo Matsushima, Shoji Oura

**Affiliations:** 1 Surgery, Kishiwada Tokushukai Hospital, Kishiwada, JPN

**Keywords:** breast cancer, cyst formation under the nipple, estrogen receptor negativity, mixed type, squamous cell carcinoma

## Abstract

It is well known that breast squamous cell carcinomas (SCCs) have highly cystic components. It, however, is not yet known whether the location of cystic areas contributes to the diagnosis of breast SCCs. A 62-year-old woman with a right breast mass was referred to our hospital. Mammography showed an oval mass just under the right nipple and distortion of the adjacent mammary gland with pleomorphic calcifications. Ultrasound showed a subnipple large oval cystic lesion with solid parts, microcalcifications, and an adjacent polygonal mass, both with a high depth/width ratio and internal punctuate high echoes. Magnetic resonance imaging (MRI) of the masses clarified the connection between the two masses at their deep borders and showed low signals on T1-weighted images and high and slightly high signals at the cyst and mass parts on T2-weighted images, respectively. Core needle biopsy of the solid mass pathologically showed atypical cells growing in tubular and cord-like fashions with connective tissue proliferation, leading to the diagnosis of scirrhous type invasive ductal carcinoma (IDC). The patient, therefore, underwent mastectomy and sentinel node biopsy, showing pathological node negativity on frozen section. Postoperative pathological study showed that the scirrhous type IDC cells were connected to pleomorphic spindle cells and further tied up to SCC cells with central cystic structures. In addition to estrogen and progesterone receptor negativity, immunostaining showed human epidermal growth factor receptor type 2 (HER2) negativity in the IDC components and equivocality in the SCC components. Thereafter, fluorescence in situ hybridization clarified no amplification of human epidermal growth factor receptor type 2 (HER2) genes in the SCC components. The patient recovered uneventfully, received dose-dense chemotherapy, and is scheduled for long-term follow-up on an outpatient basis. Diagnostic physicians should consider mixed-type breast SCCs when breast tumors have cystic structures just under the nipple and an adjacent solid mass.

## Introduction

The mammary gland is a glandular tissue, which therefore makes breast carcinomas overwhelmingly adenocarcinomas. The mammary gland, however, can extremely rarely develop squamous cell carcinomas (SCCs), one phenotype of metaplastic carcinomas [1]. SCCs are classified into pure and mixed subtypes. The former has more than 90% SCC components, and the latter has predominant SCC components and more than 10% of invasive ductal carcinoma (IDC) components. Although many breast cancers are sensitive to chemotherapy, breast SCCs hardly respond to any type of systemic therapies [2,3]. Quite a few oncologists, therefore, have experienced great difficulty when treating metastatic breast SCCs.

In the past three decades, operable breast cancers have often come to be treated not with primary surgery but with neoadjuvant chemotherapy (NAC), based both on their biology [4-6] and patients’ preference for breast conservation. Breast SCCs are basically triple-negative breast cancers [2,3] and, especially in mixed-type SCCs, can be improperly treated with NAC because of the frequent diagnosis of estrogen receptor-negative IDC by the pathological evaluation of core needle biopsy specimens.

It is well known that approximately half of breast SCCs have cystic components within them due to their aggressive nature them. Therefore, the presence of cystic components can contribute to the diagnosis of breast SCCs to some extent. No studies, however, have clarified where cystic areas occur within SCCs and how the location of cystic areas contributes to the diagnosis of SCCs.

We herein report a breast SCC case with cystic components just under the nipple, which highly suggests that subnipple cyst formation is an important diagnostic clue for mixed-type breast SCCs.

## Case presentation

**Figure 1:**
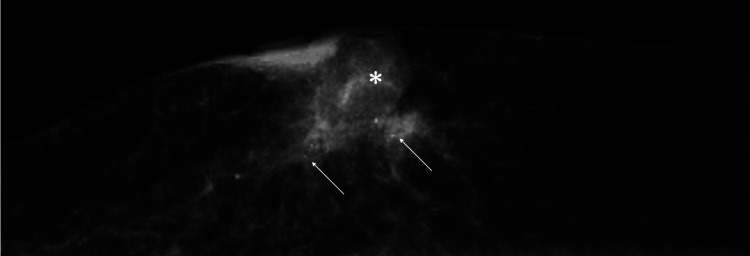
Mammography Findings Mediolateral oblique view mammography showed a sub nipple mass (asterisk) and distortion of the adjacent mammary gland (arrows) with microcalcifications.

A 62-year-old woman without any prior breast symptoms or a history of screening mammography evaluation, noticed a breast mass and was referred to our hospital due to her right breast mass. Mammography showed an oval mass just under the right nipple and distortion of the adjacent mammary gland with pleomorphic calcifications (Figure 1).

**Figure 2:**
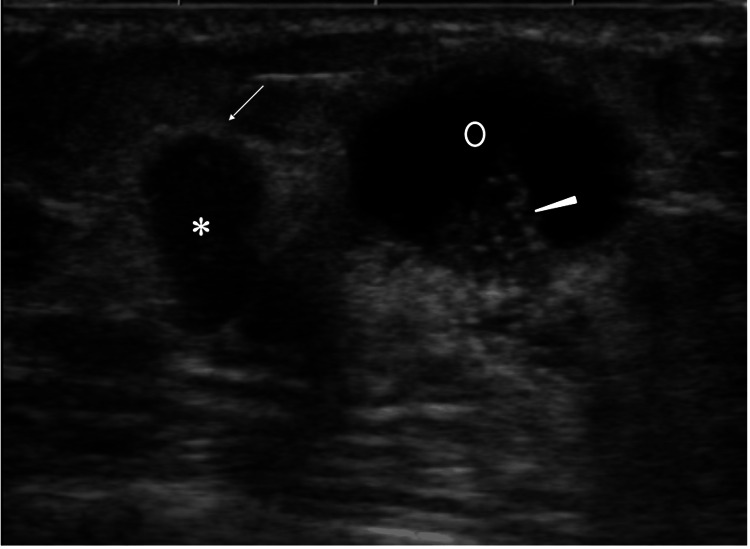
Ultrasound Findings Ultrasound showed a high depth/width ratio mass (asterisk) both with haloes (arrow) and internal punctuate high echoes, and a mixed cystic (open circle) and solid (arrowhead) mass in the nipple direction with microcalcifications in its solid parts.

Ultrasound showed a polygonal mass with a high depth/width ratio and internal punctate high echoes and an adjacent subnipple large oval cystic lesion, which had solid parts with microcalcifications (Figure 2).

**Figure 3:**
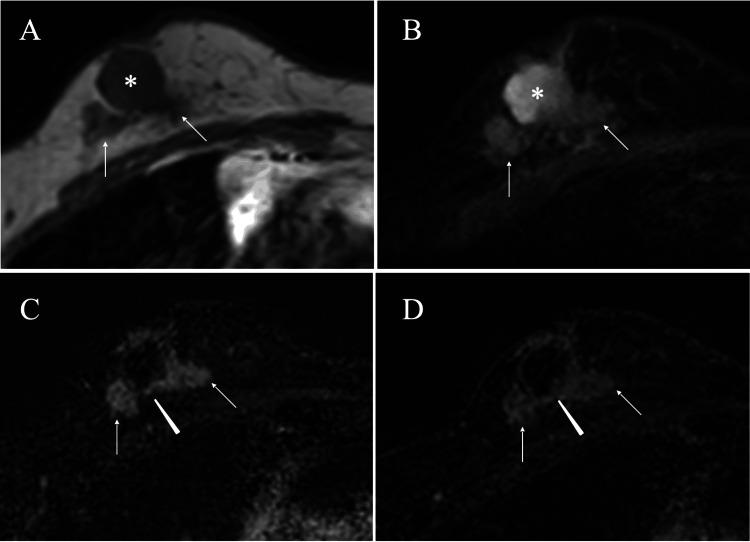
Magnetic Resonance Imaging (MRI) Findings MRI of the solid parts in the two masses showed low signals on T1-weighted images (A, arrows) and weak high signals on T2-weighted images (B, arrows). MRI of the cystic parts showed low signals on T1-weighted images (A, asterisk) and high signals on T2-weighted images (B, asterisk). Time-signal intensity images showed early enhancement on early phase images (C, arrows), retained weak enhancement on late phase images (D, arrows), and connection of the two masses at their deep borders (C and D, arrowhead).

Magnetic resonance imaging (MRI) of the masses showed a maximum size of 3.5 cm, low signals on T1-weighted images, and high and slightly high signals at the cyst and mass parts on T2-weighted images, respectively. In addition, time-signal intensity images showed weak early and retained enhancement up to the late phase, and clarified the connection between the two masses at their deep borders (Figure 3).

**Figure 4:**
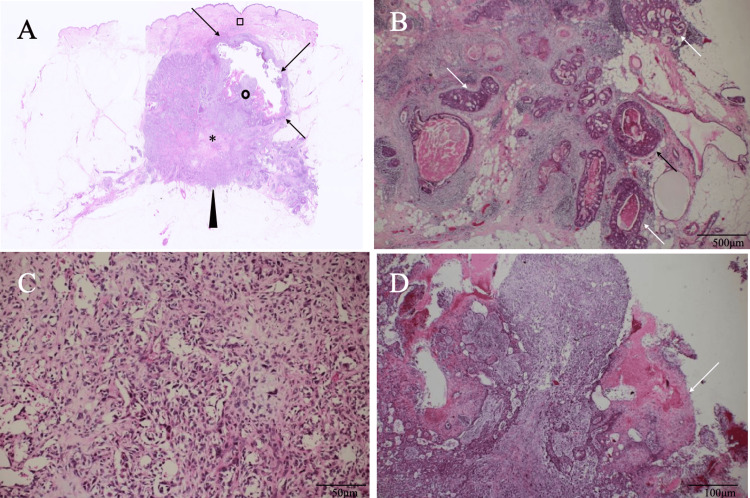
Pathological findings A) Low magnified view showed a mixed cystic mass (arrows) with solid parts (open circle) just beneath the nipple (open square) and an adjacent solid mass (arrowhead) with abundant fibrous components (asterisk). B) Magnified view of the solid mass showed atypical cells growing in a cribriform fashion (arrows). C) Spindle cells with sarcomatoid features intervened between the cystic and invasive ductal carcinoma areas. D) Solid parts in the intracystic mass had squamous cell carcinoma cells with keratinization (arrow).

These imaging findings made us do the core needle biopsy of the solid breast mass under the tentative diagnosis of breast cancer. Pathological study showed atypical cells growing in tubular and cord-like fashions with connective tissue proliferation and focal microcalcifications, leading to the diagnosis of scirrhous type IDC. Immunostaining of cancer cells showed estrogen and progesterone receptor negativity, human epidermal growth factor receptor type 2 (HER2) equivocality, and a high Ki-67 labelling index of 30%. The patient, therefore, underwent mastectomy and sentinel node biopsy, showing pathological node negativity on frozen section. Postoperative pathological study showed that the scirrhous type IDC cells were connected to pleomorphic spindle cells and further tied up to SCC cells containing central cystic structures (Figure 4).

Immunostaining showed HER2 negativity in the IDC components and equivocality in the SCC components. Thereafter, fluorescence in situ hybridization clarified no amplification of HER2 genes in the SCC components. The patient recovered uneventfully, was discharged on the seventh day after the operation, received dose-dense chemotherapy consisting of four cycles of epirubicin and cyclophosphamide followed by four cycles of paclitaxel, and is scheduled for long-term follow-up on an outpatient basis.

## Discussion

Various breast disorders can have cystic structures just beneath the nipple: intraductal papillomas, nipple adenomas, malignant phyllodes tumors, and non-invasive/IDCs growing in tubule-forming fashions [7,8]. Among these diseases, only (non-) IDCs can have microcalcifications in and around the cystic walls. MRI and ultrasound findings, therefore, made us speculate that IDCs had caused both internal punctate high echoes in the solid mass and cyst formation under the nipple. We further speculated that, probably due to the tumor heterogeneity, fibrous components present among IDC cells led to the formation of the high depth/width ratio solid mass [9].

When evaluating mixed cystic and solid breast lesions, it is naturally imperative for diagnostic physicians to obtain biopsy samples from the solid areas, which generally harbor more important pathologic elements. We, therefore, performed a core needle biopsy of the solid mass and obtained pathological information of IDC cells with estrogen receptor negativity, a high value of Ki-67 labelling index, and HER2 equivocality. It is well known that estrogen receptor (ER)-negative IDCs, regardless of HER2 status, show higher pathological complete response rates to NAC than ER-positive IDCs [10]. We, therefore, might have applied NAC to the patient if the patient had strongly requested us to treat her breast cancer with breast-conserving therapy. Breast SCCs, however, hardly respond to any type of chemotherapy and generally show progression when treated with NAC under the tentative diagnosis of ER-negative IDC.

The vast majority of tubule-forming IDCs with cystic structures can spread beyond the cystic walls, but hardly form a solid mass with abundant fibrous components [7]. Conversely, solid-type IDCs can often have ductal spread predominantly toward the nipple direction, but extremely rarely have cyst-forming type ductal spread. In short, by considering the relative positions among the solid mass, cystic parts, and the nipple, we could have negated both intracystic breast cancer with peripheral solid type invasion and solid type IDCs with cyst-forming type ductal spread toward the nipple.

It is well known that pulmonary SCCs often present cavitation due to both central necrosis and subsequent subclinical expectoration of necrotic debris via the bronchi [11]. Breast SCCs grow similarly and often have cystic components due to central necrosis. In hindsight, we should have speculated that breast cancers with both subnipple cystic parts and adjacent IDC parts could present with imaging findings similar to those observed in this case. In any case, when diagnosing an intracystic tumor just beneath the nipple and an adjacent solid tumor, diagnostic physicians need to keep in mind the possibility of mixed-type SCCs.

## Conclusions

Mixed-type SCCs can have cystic structures just beneath the nipple. Diagnostic physicians, therefore, should take mixed-type breast SCCs into mind when breast tumors have cystic structures just under the nipple and an adjacent solid mass. In addition, even if breast cancer with these structures is diagnosed as an ER-negative IDC, oncologists should be cautious about applying NAC to it.

## References

[1] Benoist P, Mureau A, Joueidi Y (2018). Management and prognosis of pure primary squamous cell carcinoma of the breast. J Gynecol Obstet Hum Reprod.

[2] Hennessy BT, Krishnamurthy S, Giordano S (2005). Squamous cell carcinoma of the breast. J Clin Oncol.

[3] Behranwala KA, Nasiri N, Abdullah N, Trott PA, Gui GP (2003). Squamous cell carcinoma of the breast: clinico-pathologic implications and outcome. Eur J Surg Oncol.

[4] Scholl SM, Fourquet A, Asselain B (1994). Neoadjuvant versus adjuvant chemotherapy in premenopausal patients with tumours considered too large for breast conserving surgery: preliminary results of a randomised trial: S6. Eur J Cancer.

[5] van der Hage JA, van de Velde CJ, Julien JP, Tubiana-Hulin M, Vandervelden C, Duchateau L (2001). Preoperative chemotherapy in primary operable breast cancer: results from the European Organization for Research and Treatment of Cancer trial 10902. J Clin Oncol.

[6] Mauri D, Pavlidis N, Ioannidis JP (2005). Neoadjuvant versus adjuvant systemic treatment in breast cancer: a meta-analysis. J Natl Cancer Inst.

[7] Oba K, Tsunoda H, Moon WK (2025). Ductal abnormalities as primary findings on breast ultrasonography: a literature review and proposed classification. Ultrasonography.

[8] Ono S, Tanaka M, Yoshinaga Y, Satou T, Aoki M (2024). A case of giant nipple adenoma. Surg Case Rep.

[9] Tsuchiya SI, Yamaguchi R, Tsuchiya K, Ohashi R (2016). Characteristics of the Japanese histological classification for breast cancer: correlations with imaging and cytology. Breast Cancer.

[10] Peto R, Davies C, Godwin J (2012). Comparisons between different polychemotherapy regimens for early breast cancer: meta-analyses of long-term outcome among 100,000 women in 123 randomised trials. Lancet.

[11] Tsao MS, Brambilla E, Nicholoson AG (2015). Squamous cell carcinoma. WHO Classification of Tumors of the Lung, Pleura, Thymus and Heart.

